# Process Study of Selective Laser Sintering of PS/GF/HGM Composites

**DOI:** 10.3390/ma17051066

**Published:** 2024-02-26

**Authors:** Lijian Liu, Shouxiao Zhu, Yongkang Zhang, Shaobo Ma, Shuxuan Wu, Bin Wei, Guang Yang

**Affiliations:** College of Mechanical Engineering, Hebei University of Science and Technology, Shijiazhuang 050018, China

**Keywords:** selective laser sintering, polystyrene, glass fiber, hollow glass microbeads, process optimization

## Abstract

To address the issues of insufficient strength and poor precision in polystyrene forming parts during the selective laser sintering process, a ternary composite of polystyrene/glass fiber/hollow glass microbeads was prepared through co-modification by incorporating glass fiber and hollow glass microbeads into polystyrene using a mechanical mixing method. The bending strength and dimensional accuracy of the sintered composites were investigated by conducting an orthogonal test and analysis of variance to study the effects of laser power, scanning speed, scanning spacing, and delamination thickness. The process parameters were optimized and selected to determine the optimal combination. The results demonstrated that when considering bending strength and Z-dimensional accuracy as evaluation criteria for terpolymer sintered parts, the optimum process parameters are as follows: laser power of 24 W, scanning speed of 1600 mm/s, scanning spacing of 0.24 mm, and delamination thickness of 0.22 mm. Under these optimal process parameters, the bending strength of sintered parts reaches 6.12 MPa with a relative error in the Z-dimension of only 0.87%. The bending strength of pure polystyrene sintered parts is enhanced by 15.69% under the same conditions, while the relative error in the Z-dimension is reduced by 63.45%. It improves the forming strength and precision of polystyrene in the selective laser sintering process and achieves the effect of enhancement and modification, which provides a reference and a new direction for exploring polystyrene-based high-performance composites and expands the application scope of selective laser sintering technology.

## 1. Introduction

Selective laser sintering technology (SLS) is based on the principle of discrete stacking. Utilizing a laser as the energy source and powder particles as the processing material, a two-dimensional model is transformed into a three-dimensional object. Under precise computer control, solid powder materials in the designated region are selectively sintered according to two-dimensional stratified cross-section information provided by the model using thermal action from the laser. The solid powder materials are then scanned layer by layer and bonded together to create 3D solid parts [1,2,3]. Compared with traditional forming methods, SLS offers advantages such as shorter production cycles, lower manufacturing costs, a wider variety of materials available for use, and no limitations on part shape during formation. This process is widely used in rapid prototyping, mold trial production and testing, new product design and development, and creative product customization [4,5].

Materials serve as the fundamental basis for 3D printing, and their properties directly impact the quality of SLS sintered parts. Polymer-based powder materials have been widely used in SLS, with polystyrene (PS) being one of the earliest and most commonly utilized matrix materials due to its advantages such as minimal shrinkage rate, high precision in forming, wide range of sintering temperatures, absence of a fixed melting point, and cost-effectiveness [6,7,8]. However, pure PS sintered parts exhibit high porosity and low densification degree, resulting in inadequate strength that significantly limits the application scope of PS powder [9]. Therefore, it is essential to conduct experimental investigations on reinforcing modifications for PS materials to establish a foundation for developing composite materials with enhanced performance. Glass fiber (GF), known for its exceptional characteristics including high mechanical strength, good chemical stability, excellent heat resistance, and affordability is frequently employed as a reinforcement material for thermoplastic resin and thermosetting resin [10]. When GF is thoroughly mixed with PS and subsequently sintered together, the forces acting on PS are effectively transmitted and dispersed onto GF. This allows the stiffness and strength inherent in GF to fortify the resulting PS sintered parts [11,12,13]. Insulating hollow glass microbeads (HGMs) possess a small expansion coefficient along with advantageous attributes like high filling capacity, good dispersion properties, and isotropy. These HGMs can reduce shrinkage rates while minimizing warpage deformation. Additionally, they enhance material anisotropy, thereby improving processing fluidity as well as dimensional stability [14]. When external forces are applied to the material, HGM absorbs energy through mechanisms such as debonding, sliding, and interlocking, resulting in deflection and passivation of cracks during expansion. This hinders crack propagation and plays a strengthening role, making it suitable for plastic reinforcement modification [15]. The synergistic enhancement of PS by these two materials effectively compensates for the limitations of single material enhancement modification and improves the quality of sintered PS parts. However, due to the distinct structure and surface properties of GF, HGM, and the organic polymer matrix, there is a slight interface compatibility issue between them. Simple blending cannot achieve uniform dispersion of inorganic fillers within the organic matrix when preparing composite materials. Therefore, prior surface treatment with silane coupling agent is necessary to enhance physical and chemical properties as well as improve dispersion within the matrix. This enhances interfacial bonding between inorganic filler and matrix [16,17].

In recent years, extensive research has been conducted by scholars both domestically and internationally on the enhancement and modification of PS. Benchouia et al. [18] prepared polystyrene/date surface fiber composites by melt blending and thermo-compression molding methods and the thermomechanical and compressive strength tests were carried out on the prepared composites, which showed that the incorporation of date surface fibers improves the thermal stability of the PS matrix, and the composites exhibit higher compressive strengths as compared to pure PS. Rodriguez et al. [19] utilized the high specific surface area of molybdenum disulfide to fabricate a polystyrene/molybde-num disulfide composite through a melt mixing process. The incorporation of 0.002 wt% molybdenum disulfide resulted in a remarkable enhancement in the tensile strength of polystyrene by 27.5%, fracture elongation by 93.7%, and toughness by 100%. Russo et al. [20] prepared polystyrene/perovskite composites by melt co-mixing method. The experimental findings demonstrated a positive correlation between the perovskite filler content and the enhanced bending properties of the composites. Yang et al. [21] fabricated polystyrene/glass fiber composite materials using the mechanical mixing method and determined the optimal glass fiber content through proportioning experiment, thereby enhancing the flexural strength of polystyrene selective laser sintered parts. Pashmforoush et al. [22] investigated the effect of calcium carbonate nanoparticles content on the properties of polystyrene and prepared calcium carbonate nanoparticles/polystyrene composites by solution blending method, which showed that the Young’s modulus, Poisson’s ratio, shear modulus, and thermal conductivity of pure PS were increased by 60.62%, 12.12%, 55.83%, and 33%, respectively, with the addition of only 7 wt% of calcium carbonate. While most scholars have focused on using a single reinforcement to enhance PS modification, resulting in limited effects, there have been a few studies exploring the use of multiple reinforcements for PS modification. The incorporation of multiple reinforcements in composites allows for the retention of each component’s advantages and the generation of synergistic effects among different parts of the material. By employing a ternary composite consisting of fibers, inorganic particles, and a polymer matrix, it is possible to achieve optimized material properties and complementary defect characteristics, leading to superior performance compared to composites reinforced with only one component. This represents an important new direction for investigating high-performance composite materials [23].

In view of this, in order to further enhance the comprehensive properties of PS matrix composites, a novel type of PS matrix composites was obtained by mechanically blending GF and HGM with PS powder and forming them through SLS. The effects of laser power, scanning speed, scanning spacing, and delamination thickness on the bending strength and Z-dimensional accuracy of sintered PS/GF/HGM composites were investigated using an orthogonal test combined with variance analysis. The variation patterns were studied to optimize the parameter combination for individual test indices. Based on this, a comprehensive analysis was conducted on the bending strength and Z-dimensional accuracy. The optimal combination of process parameters was determined to achieve the best enhancement and modification effect, providing a reference and foundation for SLS research on modified PS powder.

## 2. The Experimental Section

### 2.1. Experimental Material and Equipment

PS powder: Industrial grade with an average particle size of 120 μm, produced by Guangdong Dongguan Xutai Plastic Raw Materials Co., Guangzhou, China.

Glass fiber: Industrial grade with an average length of 74 μm and a single filament diameter of 13 μm, produced by Chongqing Heyuan Composite Materials Co., Chongqing, China.

Hollow glass microbeads: H38, with a size of 38.5 μm, produced by China Science and Technology Research Institute Co., Shanghai, China.

Silane coupling agent: KH550, produced by Quanzhou Kangjin New Material Technology Co., Quanzhou, China.

Oxalic acid: analytically pure, produced by Suzhou Haolong Chemical Co., Suzhou, China.

Analytical balance: LC-FA3204, produced by Shaoxing Shangli Instrument Co., Shaoxing, China.

Vacuum drying oven: DZF-6210BZ, produced by Chongqing Yingzhanda Instrument Co., Chongqing, China.

High speed mixer: GH-10A, produced by Beijing Plastics Machinery Factory, Beijing, China.

Selective laser sintering forming machine: AFS-500, produced by Beijing Longyuan Automatic Forming System Co., Beijing, China.

Multi-functional statics experimental machine: UTM6503, produced by Shenzhen Sansheng Technology Co., Shenzhen, China.

Field emission scanning electron microscopy: S-4800-I, produced by HI-TACHI, Tokyo, Japan.

### 2.2. Preparation of PS/GF/HGM Composite Powder

First of all, 95% of the mass fraction of ethanol aqueous solution was configured. At the same time, oxalic acid was used to adjust the pH of the solution to approximately 3, weighing GF dosage of 1.5% of the silane coupling agent KH550 poured into the appropriate amount of ethanol aqueous solution. After stirring for 10 min, the mixture was left undisturbed for 30 min to ensure thorough mixing. Subsequently, the GF was added to the prepared solution and stirred for 40 min. It was then removed, allowed to stand at room temperature for 12 h, and finally placed in a vacuum drying oven at 100 °C for 6 h before being ground and screened. The same method and steps were followed for surface coupling treatment of the HGM.

According to the literature review and preliminary material group distribution ratio experiments, the optimal sintering quality of PS/GF/HGM terpolymer is achieved when the mass ratio is 92:6:2. Using an electronic balance, PS powder was mixed with modified GF and HGM in a high-speed mixer at a mass ratio of 92:6:2 for 30 min at a speed of 1000 r/min. After thorough mixing, the powder was sieved using a vibrating screen with a mesh size of 150 μm to obtain PS/GF/HGM ternary composite material that meets the size requirements for the SLS process.

### 2.3. Preparation of Sample

The sintering experiment of PS/GF/HGM composite was conducted using the AFS-500 selective laser rapid forming machine by Beijing Longyuan Automatic Forming System Co. The AFS-500 molding machine adopts C-55L carbon dioxide laser with auto focus function: the laser wavelength is 10.6 μm; the laser spot diameter is 0.32 mm; the maximum output power is 55 W; the mode quality is 1.2 M^2^; the beam dispersion is 7.5 mard full angle; the delamination thickness is 0.08~0.35 mm; the maximum scanning speed is 6 m/s; and the molding speed is 150~400 cm^3^/h.

Sintering experiments were carried out on the prepared PS/GF/HGM composites by adjusting the laser power, scanning speed, scanning spacing, and delamination thickness at a preheating temperature of 91 °C with no support and constant alternating scanning mode in the XY direction. Five standard bending strength test samples measuring 80 mm × 10 mm × 4 mm were simultaneously sintered at the same spacing. After completion of sintering, the samples were cooled to room temperature before being removed and labeled after removing any excess floating powder on the surface.

### 2.4. Performance Testing and Characterization

Bending strength test: Three-point bending strength test using multifunctional static tester for machine PS/GF/HGM sintered parts. The test was performed at a speed of 2 mm/min, with a span of 64 mm and support rolls and applying rolls having a radius of 5 mm each. The statement is based on the principles of Formula (1). The maximum bending stress was measured to determine the average bending strength of five samples sintered simultaneously [24].

(1) $$\sigma =\frac{3FL}{2bh^{2}}$$

where σ—bending strength of sintered parts, MPa; F—maximum bending stress of sintered parts, N; L—three-point bending test scale, mm; b—width of sintered parts, mm; h—height of sintered parts, mm.

Z-direction dimensional accuracy test: The Z-direction height of the curved sample is measured to determine the dimensional accuracy of the sintered part. Five measurements are taken at equal intervals using a vernier caliper, and their average value represents the size of the sample. The test results represent the mean Z-direction height measurement for five samples sintered simultaneously, and relative size error can be calculated using Formula (2) [25].

(2) $$\delta =\frac{A_{1}-A_{0}}{A_{0}}\times 100\%$$

where δ—relative error of size; $A_{1}$—measured dimensions of sintered parts, mm; $A_{0}$—theoretical dimensions of sintered parts, mm.

SEM analysis: The prepared ternary composite powder sintered parts were mechanically fractured, followed by vacuum gold spraying treatment on the cross section for observation of the internal structure using scanning electron microscopy and photography.

## 3. Results

Based on a substantial number of previous tests conducted on PS/GF/HGM composites using SLS, while maintaining a preheating temperature of 91 °C and an unchanged XY direction alternating scanning mode, the fundamental formation of ternary composites is ensured. A more optimal combination of process parameters is obtained, encompassing a laser power of 27 W, scanning speed of 1800 mm/s, scanning spacing of 0.20 mm, and delamination thickness of 0.20 mm. The impacts of laser power, scanning speed, scanning spacing, and delamination thickness on the flexural strength and Z-dimensional accuracy of PS/GF/HGM composites are discussed and analyzed through orthogonal experiments.

The orthogonal test is a scientific research method aimed at conducting multi-factor and multi-level experiments. The selected experimental points are characterized by “uniform dispersion and precise comparability”, which not only significantly reduces the number of tests, but also achieves superior test results [26]. Bending strength and Z-dimensional accuracy were chosen as measurement indices. The laser power, scanning speed, scanning spacing, and delamination thickness were selected as the test variables, among which the laser power ranged from 24 W to 33 W, scanning speed ranged from 1600 mm/s to 2200 mm/s, scanning spacing ranged from 0.15 mm to 0.24 mm, and delamination thickness ranged from 0.16 mm to 0.22 mm. Four equally spaced level values were selected for each test variable, resulting in the design of an “orthogonal test table with four factors and four levels” as shown in Table 1 to investigate the optimal combination of laser power (A), scanning speed (B), scanning spacing (C), and delamination thickness (D). The orthogonal test scheme and its corresponding results are presented in Table 2. As observed from the orthogonal test results in Table 2, the relative error of Z-dimension for PS/GF/HGM composite sintered parts ranges from −0.67% to 7.93%, while bending strength ranges from 3.65 MPa to 12.06 MPa.

## 4. Discussion

### 4.1. Analysis of Bending Strength Variance

For orthogonal tests, analysis of variance is commonly employed to analyze and process experimental data. The greater the variance in test results under the same test factor, the stronger the influence of that factor on the test index, indicating its higher importance [27]. Variance analysis can be utilized to determine the degree of influence different process parameters have on bending strength of sintered parts, enabling optimization and identification of an optimal combination of process parameters. The analysis of variance of the bending strength of composite sintered parts was calculated according to Formulae (3)–(6) as shown in Table 3 [28], with corresponding variance analysis shown in Table 4 based on these calculations.

(3) $$m_{ij}=\frac{k}{n}M_{ij}$$

(4) $$R_{j}=\underset{1\leq i\leq j}{\max}\left\{m_{ij}\right\}-\underset{1\leq i\leq j}{\min}\left\{m_{ij}\right\}$$

(5) $$S_{j}=\frac{k}{n}\sum_{i=1}^{n}M_{ij}^{2}-\frac{1}{n}T^{2}$$

(6) $$T=\sum_{i=1}^{n}Z_{i}$$

where $M_{ij}$—sum of test indicators at the ith level of the test factor in column j; $m_{ij}$—mean value of the test indicator at the ith level of factor j; k—level number; n—number of tests; $R_{j}$—column j range; $S_{j}$—sum of squares of variance at different levels of test factor in Column j; T—measure the sum of test indexes; $Z_{i}$—measured values of test indicators per test number.

**Figure 1:**
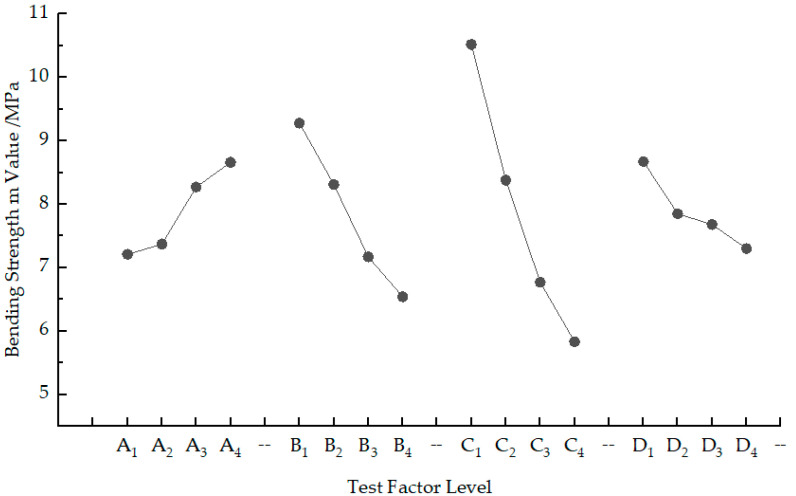
The relationship between the flexural strength of sintered parts and the level of each factor.

The relationship curves between the test factors and the factor levels for the bending strength of sintered parts of PS/GF/HGM composites were obtained by taking the levels of the test factors in the orthogonal test as the horizontal coordinates, and the m-values of their corresponding factor levels as the vertical coordinates, as shown in Figure 1.

Using sintered parts bending strength as a measure, the larger the test result value was, the higher the bending strength and the more superior the mechanical properties of the sintered parts were. Based on Table 3 and Table 4, variance calculations reveal that S_C_ > S_B_ > S_A_ > S_D_. Consequently, it can be inferred that the order of process parameters in terms of their impact on bending strength is C, B, A, and D, respectively, with scanning spacing having the greatest influence while delamination thickness has a minimal impact; scanning speed and laser power fall between these two extremes. According to Table 3 and Figure 1, A_4_ is the optimum choice for laser power A. As the laser power decreases, the laser energy received by the powder diminishes, resulting in a decrease in melting of powder particles. Consequently, there is a reduction in fusion degree between powder particles, deterioration of bonding effect, increase in internal porosity of formed parts, decrease in densification degree, and decline in bending strength. Scanning speed B is best at B_1_; when scanning speed increases, the energy per unit time for laser scanning on the powder decreases. This leads to insufficient melting of the powder itself with minimal changes to its melting state [29]. Inadequate wetting and diffusion occur as well as reduced sintering neck formation between adjacent powder particles which ultimately results in the decreased bending strength of sintered parts. C_1_ represents the optimal scanning distance C; with increased scanning distance comes a decrease in the number of laser scans and overlap between adjacent scan lines. This leads to lower bonding rates where only surface particle fusion bonding occurs instead of full fusion bonding between powders [30]. Consequently, connection strength between scan lines decreases along with corresponding reduction in bending strength [31]. The preferred delamination thickness D is D_1_; increasing delamination thickness causes a decrease in absorbed energy by the powder per unit area, leading to inadequate deep sintering due to insufficient laser energy input. Partial melting occurs, resulting in weak bonds between layers causing lower connection strength during the sintering process and subsequently a reduction in bending strength [32]. Therefore, when considering the bending strength as the measurement index, the optimal level of bending strength for sintered parts is A_4_B_1_C_1_D_1_. In other words, the ideal combination of process parameters is a laser power of 33 W, scanning speed of 1600 mm/s, scanning spacing of 0.15 mm, and delamination thickness of 0.16 mm. This parameter combination aligns with Group 1 in the orthogonal test table and does not require any additional testing. At this point, the bending strength of the sintered part reaches 12.06 MPa.

### 4.2. Precision Analysis of Variance

**Figure 2:**
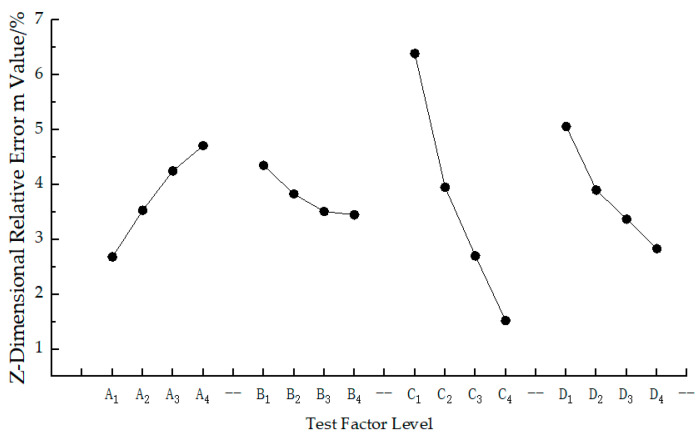
The relationship between the relative error of the Z-dimension of sintered parts and the level of each factor.

The test data analysis and calculation results of the Z-dimensional relative error of sintered parts are presented in Table 5, while the variance analysis results of the Z-dimensional relative error are shown in Table 6. Figure 2 illustrates the relationship curve between the relative error of the Z-direction size of sintered parts and various factor levels. The calculation methods and steps for each data in Table 5 remain consistent with those in Table 3. Taking the relative error of Z-direction dimension of sintered parts as a measure, a smaller test result value indicates higher dimensional accuracy of the sintered part. Based on Table 5 and Table 6, from the results of the variance calculation is S_C_ > S_D_ > S_A_ > S_B_. Therefore, it can be concluded that the degree of influence of each process parameter on the relative error of Z-direction dimensions is C, D, A, and B in descending order; where scanning spacing has the greatest impact while scanning speed has minimal impact, with delamination thickness and laser power falling between these two factors. According to Table 5 and Figure 2, A_1_ is the optimal choice for laser power A. If the laser power is increased, the energy exerted on the powder bed surface will be increased, leading to an expansion of its heat-affected zone [33]. Consequently, the surrounding powder near the sintered part melts and adheres together, resulting in a secondary sintering phenomenon and an increase in dimensional errors of the formed part [34]. The best scanning speed B is B_4_. As the scanning speed decreases, it prolongs the scanning time of the laser on each powder layer. This leads to a higher absorption of energy by the powder, causing more complete melting and increased shrinkage deformation as well as gradual growth in size errors [35]. C_4_ is identified as being most suitable for scanning spacing C. Decreasing this interval enhances overlap between adjacent scanning lines which results in repeated sintering of powder within these areas forming larger cooling shrinkage regions that contribute to dimensional errors [36]. The ideal delamination thickness D is D_4_. Reducing delamination thickness D strengthens laser penetration into powders during sintering process; however, excessive deep sintering may occur, particularly at bottom layers due to repeated exposure resulting from stronger penetration ability thereby increasing Z-dimension error [37]. Consequently, when considering the relative error of the Z-dimension as the measurement index, the optimal level of Z-dimensional accuracy for PS/GF/HGM terrains composite powder SLS sintered parts is A_1_B_4_C_4_D_4_. In other words, the optimal combination of process parameters is a laser power of 24 W, scanning speed of 2200 mm/s, scanning spacing of 0.24 mm, and layer thickness of 0.22 mm. This parameter combination aligns with Group 4 in the orthogonal test table and does not require any additional testing. At this stage, the relative error in the Z-dimension for sintered parts is −0.67%.

### 4.3. Determine the Best Process Parameter Combination

The optimal combination of process parameters for achieving high bending strength is determined as A_4_B_1_C_1_D_1_, while the optimal combination for ensuring Z-direction dimensional accuracy is obtained as A_1_B_4_C_4_D_4_. It can be observed that bending strength and dimensional accuracy are two contrasting performance indicators. To simultaneously achieve both strength and accuracy, the Formula (7) of m-value change rate K is introduced for further optimization analysis. This is to ensure that one index is better, while the other is less variable, to achieve the optimum combination of process parameters, taking into account both strength and precision.

(7) $$K=\frac{\left|m_{i}-m_{j}\right|}{m_{i}}$$

Formulas containing: K—m-value change rate; $m_{i}$—the m-value of a factor at an i level; $m_{j}$—the m-value of a factor at a j level.

The bending strength is maximized when the laser power is set to A_4_, which serves as the evaluation criterion. Furthermore, an analysis of dimensional accuracy in the Z-direction reveals that selecting laser power A_1_ results in minimal dimensional errors. The value changes from A_1_ to A_4_ with a change rate of 0.75. In terms of bending strength analysis, there is a change from A_4_ to A_1_, with a change rate of 0.17. Therefore, choosing laser power A_1_ ensures good Z-direction dimensional accuracy for PS/GF/HGM composite sintered parts while maintaining little variation in bending strength. Similarly, scanning speed B_1_, scanning spacing C_1_, and delamination thickness D_1_ were selected as indices for evaluating bending strength. The corresponding change rates for Z-direction dimensional accuracy were 0.25, 3.59, and 0.79, respectively. For evaluating Z-direction dimensional accuracy, scanning speed B_4_ was chosen along with scanning spacing C_4_ and delamination thickness D_4_ as indices. The corresponding change rates for bending strength were 0.27, 0.45, and 0.16, respectively. Therefore, the scanning speed is selected as B_1_, the scanning spacing is selected as C_4_, and the delamination thickness is selected as D_4_.

After a comprehensive analysis of the bending strength and accuracy, the optimal process parameter combination for PS/GF/HGM composite sintered parts is determined to be A_1_B_1_C_4_D_4_, which corresponds to a laser power of 24 W, scanning speed of 1600 mm/s, scanning spacing of 0.24 mm, and delamination thickness of 0.22 mm. Subsequently, SLS tests were conducted on both pure PS powder and PS/GF/HGM composites using this specific set of process parameters. The resulting bending strength measurement for the sintered PS/GF/HGM composites was found to be 6.12 MPa with a relative error in Z-direction of only 0.87%. Notably, compared to sintered pure PS samples, the bending strength exhibited an impressive increase of 15.69%, while simultaneously reducing the relative error in Z-dimension by an impressive margin of 63.45%.

### 4.4. Microstructure of Sintered Parts

**Figure 3:**
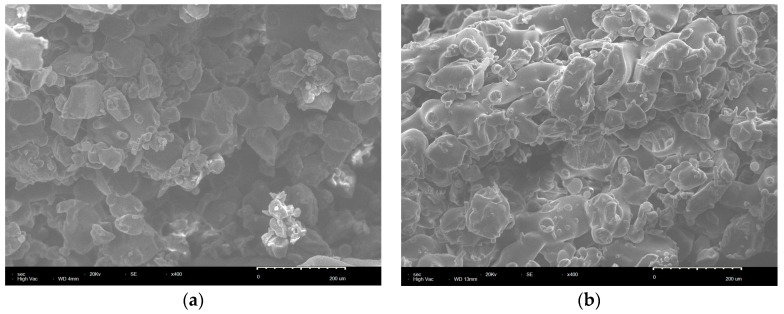
SEM image of SLS sintered bending specimen section. (**a**) The cross-section of the pure PS bending specimen is enlarged by 400 times. (**b**) The cross-section of the PS/GF/HGM composite bending specimen is enlarged by 400 times.

**Figure 4:**
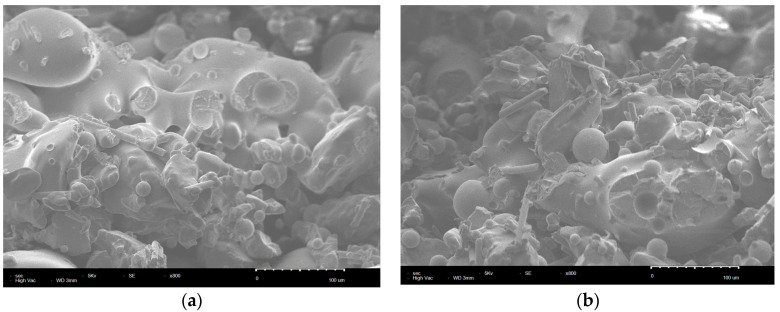
SEM image of PS/GF/HGM composite bending specimen is enlarged by 800 times. (**a**) Bridging of GF in PS/GF/HGM composites. (**b**) Desticking of HGM in PS/GF/HGM composites.

The cross-sectional morphology of the bent sample of pure PS and PS/GF/HGM composites sintered under the optimal combination of process parameters was observed using scanning electron microscopy. Figure 3 illustrates the microstructure of the bending specimen of pure PS and PS/GF/HGM composite material at 400× magnification. As depicted in Figure 3a, the cross-sectional morphology of the pure PS bending specimen appears relatively flat, with numerous irregularly shaped cavity structures within the sintered part indicating insufficient powder melting. Additionally, only limited areas exhibit sintering neck formation upon contact, which is small in size and results in poor bonding performance between powders leading to reduced strength [38]. The section of the bent sample of PS/GF/HGM composite material, as depicted in Figure 3b, exhibits a relatively rough surface. This can be attributed to the incorporation of GF and HGM, which effectively restricts the rapid projection of laser energy in the Z-direction. Consequently, more energy is absorbed by PS, leading to enhanced fusion among powder particles and an improved powder bonding effect. Moreover, the presence of GF and HGM impedes crack propagation by inducing deflection and passivation mechanisms, so that the specimen appears rough [39,40]. In comparison with pure PS sintered parts, the sintering neck formed through particle adhesion within PS/GF/HGM composites is noticeably larger and even displays a visco-fluid appearance. Additionally, these composites exhibit fewer pores and higher densification degree. Notably, both GF and HGM are uniformly dispersed within the PS matrix without agglomeration issues; furthermore, the orientation of GF appears random [41]. The microstructure of the PS/GF/HGM composite bending sample under the optimal combination of process parameters at 800 times magnification is depicted in Figure 4. The PS matrix effectively coats the GF and HGM, ensuring their embedding within the matrix and their strong adhesion to it. Additionally, a small number of GF and HGM are exposed outside the PS matrix, contributing to improved powder bed density by filling pores between PS particles due to their small particle sizes [42]. Importantly, no phase change occurs between the GF and HGM during sintering, minimizing warping in the final parts. When subjected to external force, stress is transmitted from the PS matrix to both GF and HGM, utilizing their strength for load bearing and dispersion. During the process, Figure 4a,b illustrate the extraction and interlocking of GF, as well as the removal of adhesion and immobilization of HGM. These procedures entail significant energy consumption and absorption, thereby diminishing the external force exerted on the matrix while effectively enhancing its strength [43,44]. As a result, the bending strength and dimensional accuracy of PS/GF/HGM composite powder sintered parts were significantly improved compared to pure PS.

## 5. Conclusions

The PS/GF/HGM composite was prepared through mechanical mixing method to enhance the sintering quality of PS powder, followed by the conducted SLS experiment. The forming process parameters of the PS/GF/HGM composite material were optimized using orthogonal test and analysis of variance. The experimental findings are as follows:

(1) Referring to bending strength as the index, the degree of influence of process parameters on PS/GF/HGM composites’ bending strength ranks from highest to lowest as scanning spacing, scanning speed, laser power, and delamination thickness. The optimal combination of process parameters is a laser power of 33 W, scanning speed of 1600mm/s, scanning spacing of 0.15 mm, and delamination thickness of 0.16 mm. At this point in time, sintered parts can achieve a bending strength of 12.06 MPa.
(2) The influence of process parameters on the relative error of Z-dimension in PS/GF/HGM composites, as measured by accuracy, follows the order of scanning spacing, delamination thickness, laser power, and scanning speed. The optimal process parameters for achieving a minimal relative error in the Z-direction size of sintered parts are as follows: laser power at 24 W, scanning speed at 2200 mm/s, scanning spacing at 0.24 mm, and delamination thickness at 0.22 mm. Consequently, the resulting relative error of Z-direction dimensions of sintered parts is −0.67% at this time.
(3) After evaluating the strength and precision of the PS/GF/HGM composite sintered component within the experimental range, the optimal process parameter combination for this part is a laser power of 24 W, scanning speed of 1600 mm/s, scanning spacing of 0.24 mm, and delamination thickness of 0.22 mm. Under these process conditions, the sintered parts exhibit a bending strength of 6.12 MPa and a relative error of Z-dimension at 0.87%, which is 15.69% higher than that of pure polystyrene powder sintered parts, while reducing the relative error by 63.45%.

However, there are certain limitations in this study. The molding quality of the composite material not only relies on the properties of the matrix and reinforcement materials, but also significantly depends on factors such as particle size, shape, and addition amount of the reinforcement material; dispersion of the reinforcement material; interface bonding strength between the matrix and reinforcement material; and thickness of the matrix between the reinforcements. Moreover, these influencing factors do not act independently but exhibit a certain level of interdependence and correlation. Therefore, in future research endeavors, it is crucial to comprehensively consider these factors affecting material-strengthening effects to further enhance laser sintering formed composite material quality.

## Figures and Tables

**Table 1:** Orthogonal test factor level table.

Level	Experimental Factor			
	Laser Power (A)/W	Scanning Speed $(B)/\mathrm{mm}\cdot s^{-1}$	Scanning Spacing (C)/mm	Delamination Thickness (D)/mm
1	24	1600	0.15	0.16
2	27	1800	0.18	0.18
3	30	2000	0.21	0.20
4	33	2200	0.21	0.22

**Table 2:** Orthogonal test scheme and test results.

Group Number	Experimental Factor				Experimental Result	
	A/W	$B/\mathrm{mm}\cdot s^{-1}$	C/mm	D/mm	Z-Dimensional Relative Error/%	Bending Strength/MPa
1	24	1600	0.15	0.16	7.93	12.06
2	24	1800	0.18	0.18	2.41	7.92
3	24	2000	0.21	0.20	1.06	5.20
4	24	2200	0.24	0.22	−0.67	3.65
5	27	1600	0.18	0.20	4.02	9.28
6	27	1800	0.15	0.22	5.98	9.84
7	27	2000	0.24	0.16	1.66	5.23
8	27	2200	0.21	0.18	2.45	5.11
9	30	1600	0.21	0.22	2.17	7.80
10	30	1800	0.24	0.20	1.82	6.47
11	30	2000	0.15	0.18	7.46	10.37
12	30	2200	0.18	0.16	5.53	8.43
13	33	1600	0.24	0.18	3.28	7.99
14	33	1800	0.21	0.16	5.12	8.96
15	33	2000	0.18	0.22	3.84	7.88
16	33	2200	0.15	0.20	6.59	9.78

**Table 3:** Bending strength test data analysis and calculation table.

Calculated Value	Experimental Factor			
	A	B	C	D
M_1_	28.83	37.13	42.08	34.68
M_2_	29.49	33.22	33.51	31.39
M_3_	33.07	28.68	27.07	30.73
M_4_	34.61	26.97	23.34	29.20
m_1_	7.2075	9.2825	10.52	8.67
m_2_	7.3725	8.305	8.3775	7.8475
m_3_	8.2625	7.17	6.7675	7.6825
m_4_	8.6525	6.7425	5.835	7.30
R_j_	1.445	2.54	4.685	1.37
S_j_	5.82650	15.78215	50.54675	4.00185
Prioritization Scheme	A_4_	B_1_	C_1_	D_1_

**Table 4:** Analysis of bending strength variance.

Variance Factor	Variance	Degree of Freedom	Average Variance	Error Ratio	Significance Level
A	5.8265	3	1.942167	19.902647	**
B	15.78215	3	5.260717	53.909991	***
C	50.54675	3	16.848917	172.661828	****
D	4.00185	3	1.33395	13.669855	*
Error Value	0.29275	3	0.097583		
Total	76.45	15			

*: Non-significant; **: significant; ***: relatively significant; ****: extremely significant.

**Table 5:** Analysis and calculation table of Z-dimensional relative error test data.

Calculated Value	Experimental Factor			
	A	B	C	D
M_1_	10.73	17.4	27.96	20.24
M_2_	14.11	15.33	15.8	15.06
M_3_	16.98	14.02	10.8	13.49
M_4_	18.83	13.9	6.09	11.32
m_1_	2.6825	4.35	6.99	5.06
m_2_	3.5275	3.8325	3.95	3.9
m_3_	4.245	3.505	2.7	3.3725
m_4_	4.7075	3.475	1.5225	2.83
R_j_	2.025	0.875	5.4675	2.23
S_j_	9.377169	1.983419	66.381019	10.883619
Prioritization Scheme	A_1_	B_4_	C_4_	D_4_

**Table 6:** Z-dimensional relative error variance analysis table.

Variance Factor	Variance	Degree of Freedom	Average Variance	Error Ratio	Significance Level
A	9.377169	3	3.125723	5.157763	**
B	1.983419	3	0.661140	1.090949	*
C	66.381019	3	22.127006	36.511825	****
D	10.883619	3	3.627873	5.986362	***
Error Value	1.818069	3	0.606023		
Total	90.443295	15			

*: Non-significant; **: significant; ***: relatively significant; ****: extremely significant.

## Data Availability

The raw data supporting the conclusions of this article will be made available by the authors on request.
